# When your goals inspire my goals: the role of effort, personal value, and inference in goal contagion

**DOI:** 10.1080/23743603.2020.1767502

**Published:** 2020-06-08

**Authors:** Katja Corcoran, Hilmar Brohmer, Lisa V. Eckerstorfer, Silvia Macher

**Affiliations:** a Institute of Psychology, University of Graz, Graz, Austria; b BioTechMed-Graz, Graz, Austria; c Rationality Enhancement Group, Max Planck Institute for Intelligent Systems, Tübingen, Germany

**Keywords:** Goal contagion, social influence, goal pursuit, inference processes, automatic goal activation

## Abstract

Just observing other people can influence what we do. Under certain conditions, it inspires us to strive for the same goal as the other person. Such goal contagion occurs, because one first automatically infers the goal and then adopts it for oneself. In a series of three experiments (overall *N* = 840 university students), we investigated personal goal value and the observed person’s effort as moderators of goal contagion, which is mediated by goal inference. In all three experiments, participants read a brief story about a student who either wants to earn money (target goal) or to do an internship (control) and expects to show much or little effort. In Studies 1a and b, goal inference was the dependent variable, whereas in Study 2, we considered the full moderated-mediation model and measured how strongly participants pursue the goal to earn money. We aimed at locating the moderators within this two-step process. We hypothesized that high effort increases goal inference, whereas personal goal value strengthens the relationship between goal inference and goal adoption. Across experiments, we did find evidence for explicit and spontaneous, but not for implicit goal inference. Furthermore, participants did not pursue to earn money to a different degree across conditions and different degrees of goal value. Taken together, neither the moderated-mediation process nor the basic goal contagion effect was supported. Results are discussed in the light of other published studies on goal contagion and the current Replication Crisis.

## Introduction

The social environment has a great impact on peoples’ behavior. Even though we tend to underestimate the influence of our social surroundings (Ross, 1977), there is plenty of evidence that people conform to the behavior of others. This influence could be based on normative or informational social influence (Cialdini, 2003), observational learning (Bandura, 1977), imitation (Meltzoff & Moore, 1989), mimicry (Chartrand & Lakin, 2013), or because others serve as inspiring role models (Morgenroth et al., 2015).

In cases of mimicry and imitation, people closely model the observed behavior. However, this is not always true. For example, in the famous Bobo-doll experiment by Bandura et al. (1963), it became clear that children not only directly imitate the aggressive behavior of the role model like kicking the doll through the room. They also showed new kinds of aggressive behavior like pointing a gun at the doll. Thus, observing another person’s behavior might increase the likelihood of showing the exact same behavior, or the likelihood of showing a somewhat related behavior. But how are these different types of behavior related to each other?

Sometimes, two different behaviors are related because both are means for the same goal. Kicking a doll and pointing a gun at the doll both indicate the intention to “harm” the doll. Observing the behavior of other people therefore might not only trigger expression of the same behavior, but also the adoption of the goal that the other person pursues with this behavior. This process has been coined goal contagion (Aarts et al., 2004).

If we think about the power of the social environment, goal contagion is interesting to consider for several reasons. First, goal contagion elicits goal-directed behavior that is highly flexible and persistent. People can adjust their behavior to reach the goal to given opportunities and circumstances (Gollwitzer & Moskowitz, 1996). Even if it is not possible to imitate the behavior of the inspirational other directly, there might be opportunities for alternative behavior to pursue the goal. Second, activated goals tend to influence behavior over a longer period. Once a goal is activated, it directs attention and behavior toward goal pursuit until it is fulfilled or suppressed by other, competing goals (Moskowitz, 2002). Finally, goal-directed behavior can occur automatically, outside of peoples’ awareness and without recruiting additional cognitive resources (Chartrand & Bargh, 1996). Thus, goal contagion is a potential tool to elicit prolonged goal-directed behavior automatically.

In an initial study on goal contagion by Aarts et al. (2004), participants read about a student who is looking forward to a vacation with his friends and uses the remaining time to either work on a farm or volunteer at a community center. Working on a farm implies the goal to earn money. Thus, when later given the opportunity to earn an extra income themselves, the participants in this condition eagerly took the opportunity. Participants were inspired by the observation of the other’s behavior and adjusted their own behavior accordingly. However, such a goal contagion process does not occur under all circumstances.

Several moderators have been identified which might strengthen or weaken goal contagion. For example, in the study on earning money (Aarts et al., 2004), only those people were infected for whom this goal had a high value because they were in a high need for money. Other moderators are for example, psychological distance (Wessler & Hansen, 2016), social acceptability (Aarts et al., 2004), group membership (Loersch et al., 2008), the effort of the observed behavior (Dik & Aarts, 2007), gratitude (Jia et al., 2015), or social power (Jia et al., 2018). In the present research, we want to take a closer look at the effects of moderators on goal contagion. In particular, we argue that there are at least two classes of moderators that unfold their effects in two distinct ways. This distinction becomes possible because goal contagion is conceptualized as a two-step process and a moderator might alter either the first, or the second step.

The first step of people is to infer a goal from the observed behavior. Research on spontaneous trait inferences and on automatic internal attribution indicate that people often, but not exclusively (Ham & Vonk, 2003), consider internal causes for other people’s behavior. These cognitive inferences occur fast, unconsciously, and without intention (Hassin et al., 2002). Because goals direct and motivate behavior (Gollwitzer & Oettingen, 2012), goals are likely internal causes. Even young children are able to automatically infer the goals from other people’s behavior (Hassin et al., 2005). Thus, when seeing the model kicking the doll, the children might automatically infer that the person’s goal is to harm the doll. The second step involves adopting the goal and pursuing it. Research on goal contagion indicates that behavior observation does not only activate the corresponding goal in the observers’ mind, it also motivates them to engage in behavior to pursue this goal themselves. Consistent with research on unconscious goal pursuit (Custers & Aarts, 2010), this step occurs in a quick and automatic fashion outside conscious awareness (Laurin, 2016).

Taken together, goal contagion describes an automatic effect of observation on goal activation or goal pursuit that is mediated by goal inference (Custers & Aarts, 2010). A moderator might therefore either influence the likelihood by which an observer infers from the behavior on the pursued goal (goal inference) or it might influence to what extent the inferred goal activates this goal as personal goal of the observer and initiates goal-directed behavior (goal adoption). So far, only very little research focuses on explaining the influence of moderators in more detail and specifying which part of the process is influenced.

One exception to this shortcoming is research on effort. Dik and Aarts (2007, 2008) demonstrate that effort does not only moderate the effect of observation on behavior, but also on goal inference. In their studies (Dik & Aarts, 2007), participants observed the movements of geometric shapes in an animated movie. The scene depicts a small ball trying to free a kite that is stuck in a tree. A big ball enters the scene and approaches a square with four closed doors containing a ladder. Effort was manipulated by the number of doors the big ball tries to open to reach the ladder in the square and the accessibility of helping related words in a word completion and a reaction time task served as indicator for automatic goal inference. As expected, the more doors the big ball tried, the more accessible helping related words were in the observers’ mind. In a third experiment, participants had indicated their willingness to help (filling out an extra questionnaire without additional payment) after watching the animated movie. Again, the more effort the big ball showed in the movie, the higher participants’ willingness to help. This series of experiments strongly implies that effort moderates the effect of goal contagion, because it moderates the first step in the process, which is goal inference.

It is less clear at which step the other moderators influence the process of goal contagion. Gratitude, for example, might increase goal inference, because “gratitude involves a heightened sensitivity toward social others’ experiences” (Jia et al., 2014, p. 749). However, gratitude might in addition strengthen goal adoption. Gratitude nurtures fruitful relationships and brings people together (Algoe et al., 2008). To ease coordinated actions, automatically adopting the goals of others would be helpful (Jia et al., 2014). Value, on the other hand, might specifically increase goal adoption. In other words, the higher the value of a goal, the more a person considers reaching the goal to be beneficial (Locke, 1969; Tang et al., 2008) and the more intensively she pursues a goal (Gollwitzer & Oettingen, 2012). For example, a person might infer the goal to earn money when observing a student who is working on a farm, but their own low need for money might prevent them to adopt this goal and act on it. Finally, socially unacceptable behavior might decrease goal contagion despite increasing goal inference. Because strong external causes like social norms seem unlikely for socially unacceptable behavior, internal causes like goals might readily be inferred. If socially unacceptable behavior leads nevertheless to little goal contagion (Aarts et al., 2004), strong effects in the opposite direction in the second step (goal adoption) have to take place. Overall, we still know very little about the mechanisms by which certain moderators shape the goal contagion process.

To disentangle an effect on goal inference from an effect on goal adoption, we need to separate the two steps of goal contagion by measuring goal inference. Unfortunately, the basic mediation model was rarely tested and no established goal inference measure within the framework of goal contagion exists yet.

One difficulty arises out of the distinction between explicit and implicit goal inference. Apparently, explicit goal inference does not necessarily mediate goal contagion. In the study by Dik and Aarts (2007), participants observed a big ball “trying to help” a small ball. Afterwards, participants indicated their willingness to fill out an extra questionnaire. To assess goal inference, they were asked whether they thought that the big ball wanted to help. Interestingly, including this explicit goal attribution measure as covariate into the analysis did not alter the effect of the video on participants’ willingness to help. Thus, goal attribution did not mediate goal contagion. Dik and Aarts (2007) conclude that such an explicit goal attribution measure might not capture the implicit goal inference that is part of automatic goal contagion.

Instead of asking people about the goal of the other person, one might incorporate an implicit goal inference measure. There is one study in which implicit goal inference seems to mediate goal pursuit. Jia et al. (2014) used the classic earning money goal and assessed participants’ accessibility of money-related words with a lexical decision task. In this study, the goal contagion effect was indeed mediated by accessibility of money-related words. However, there are downsides to using implicit measures when trying to capture goal inference.

Implicit measures like lexical-decision tasks or word completion tasks (Dik & Aarts, 2007; Jia et al., 2014) do not differentiate between goal inference, goal activation/accessibility, and construct accessibility. Even though goal inference necessarily leads to goal accessibility (if one infers that another person works to pursue the goal of earning money, the goal of earning money will become accessible), goal inference refers to the goal of the *other* person, whereas goal accessibility does not differentiate between personal goals or goals of other people. Therefore, it is not surprising that in the framework of goal contagion, goal accessibility is sometimes used to measure goal adoption (e.g. Jia et al., 2014, Study 3) and sometimes used to measure goal inference (Dik & Aarts, 2007, Experiment 1). The differentiation between goal accessibility and construct accessibility is also fuzzy. If a goal is activated, the corresponding construct is accessible (Kruglanski et al., 2002): an active goal of earning money should make words like “money,” “wealth,” “rich” more accessible. However, such words would also be more accessible if one merely thinks about the construct money, without having the actual goal to earn money. Thus, measures like lexical decision tasks or word completion tasks might have the advantage to be implicit, but they have the disadvantage to be less diagnostic for goal inference.

Another difficulty of measuring goal inference as mediator for the automatic goal contagion process is the point of measurement. Ideally, one would want to assess goal inference between manipulating the observed behavior and measuring goal adoption because mediation is inherently causational (Baron & Kenny, 1986). However, explicit goal inference measures could easily interrupt or disturb the automatic process, because participants would explicitly think about the goal of the observed person, or it would at least shift the process into consciousness. Therefore, such measures have to be conducted after goal adoption. It seems to be less risky to implement implicit measures at the point of mediation within the process. However, depending on the task, the implicit measurement might potentially prime a certain construct and thereby reduce or even override an effect of the goal manipulation on goal adoption. For example, if all participants work on a lexical decision task including several words related to money, the construct money might be primed by the task and influence subsequent behavior (Weingarten et al., 2016). Thus, neither explicit nor implicit measures of goal inference are ideal to capture goal inference as mediator of the automatic goal contagion process. Even though this is not the main purpose of the present research, we will address this issue and explore several measurements.

Our main goal is to understand better under which circumstances people are inspired by others, take over their goals, and start pursuing these goals themselves. Therefore, we have to acknowledge the complexity of this process. Goal contagion could be powerful, because it unfolds automatically and results in flexible and persistent goal pursuit. However, it is also vulnerable. Two crucial steps are necessary, goal inference and goal adoption, and both could be interrupted. We assume that some moderators mostly influence the first step, whereas others unfold their effect at the second step.

To shed light on this issue, we will focus on two moderators: effort and goal value. Effort is an expression of how much value the observed person places on the goal, whereas goal value focuses on the observer’s perspective. Both moderators should make a goal more contagious, but the precise mechanisms might differ. The effort the observed person puts into a behavior shall make goal inference more likely. As has been demonstrated by Dik and Aarts (2007), the manipulation of effort facilitates goal inference. However, once the goal is correctly inferred, effort might have little effect on goal adoption. On the other hand, the value of a certain goal might strengthen the goal adoption step because it accurately represents something that the observer considers to be beneficial and valuable. However, the value the observer sees in a goal might have little influence on the inference of the other person’s goal from his or her behavior (Aarts et al., 2004, Study 6). Thus, even though effort and personal goal value both moderate goal contagion, they might do so in a very different way.

A second aim of our research is to provide empirical evidence for the assumed two-step model and the mediation via goal inference. We assess automatic goal inference and include it into the statistical model in order to differentiate between moderators that alter the effect of the observed behavior on goal inference (like effort) and moderators that alter the effect of goal inference on goal adoption (like goal value). In our research, we will explore and combine several methods to measure automatic goal inference.

## Study 1a: influence of behavior observation and effort on goal inference

In the first study, we focused on the first step of goal contagion: goal inference. Similar to the classic study by Aarts et al. (2004), we manipulated the implied goal of the observed behavior by either describing the protagonist as planning to work (implying the goal to earn money) or to volunteer. In this study, we wanted to ensure that the behavior descriptions indeed produce the implied goal inferences. In addition, we manipulated effort as the crucial moderator in this step. If somebody is planning to work, an observer should infer the goal of earning money and especially so, if the protagonist shows high effort. We also included two measures for goal value, even though we did not expect an effect on goal inference. We used *need for money* as situational goal value like in Aarts et al. (2004) and we added *greed for money* (see Seuntjens et al., 2016) to take dispositional goal value into account. However, we only used those measures for exploratory analyses.

### Method

We preregistered this and the following two studies in the Open Science Framework (OSF): https://osf.io/ef59b/. All materials and data are also accessible via this link. This study was approved by the university’s ethics committee and written informed consent was obtained of each participant in compliance with the principles contained in the European Federation of Psychologists’ Associations Meta Code of Ethics and in accordance with the General Data Protection Regulation (EU) 2016/679 (this procedure was identical for all other studies mentioned in this article).

#### Design, power, and sample

We realized a 2 (goal: earning money vs. control) × 2 (effort: high vs. low) between-subjects design. Sample size was determined a priori (Faul et al., 2007), assuming power = 80%, *α* err probability = .05 and a small effect of *r* = .230 (*f* = .236),^1^ which yielded *N* = 151. The effect estimation was based on the smallest reported effect of effort on goal inference (Dik & Aarts, 2007). To account for potential dropouts, we decided to increase the sample size to *N* = 160. We recruited students of the University of Graz on campus and randomly allocated them to one of the four conditions. All material was presented on paper. Participants’ mean age was 22.35 years (*SD *= 2.78) and 57.5% were female.

#### Materials

To manipulate the goal, we presented participants with a text about a same-sex student who was planning to either work in a supermarket (goal: earning money) or to volunteer as an intern in a community center (control). In the high effort condition, the student expected to invest extra time in evenings and on weekends, which is out of the question in the low effort condition. The texts can be found in the OSF. Previously to this study, we had tested other versions of these texts, which were more closely modelled after Aarts et al. (2004) and contained information about an upcoming vacation with friends. Because an insufficient number of participants in our sample inferred the goal of earning money if the student was planning to work in the supermarket (36%; *N *= 327; further information in the “pilot study” folder in the OSF), we omitted direct references to alternative goals (like going on a vacation or spending time with friends).

Directly after reading the texts, we administered the goal inference task. Based on the assumption that explicit goal attribution is a stronger indicator of automatic goal inference if people spontaneously perceive and report this goal, this task consisted of two components. First, participants answered an open *spontaneous goal inference* question (“What do you think Stefanie is trying to achieve?”). Answers were coded with 2 if indicating the goal of earning money and with 1 if not. Second – on the next page – we listed the target goal (earning money) and four distractors (job experience, self-fulfillment, life satisfaction, and work–life balance). Participants rated the likelihood by which the student pursued each of these goals (*explicit goal inference*, 1 *not at all likely* to 9 *very likely*). By multiplying spontaneous with explicit goal inference for the target goal, we created a measure for *automatic goal inference*,^2^ in which the spontaneous measure served as a weight for the explicit measure.

Next, we measured perceived effort with two questions (*r*(160) = .49; e.g. “How much effort will the work require?”; 1 *not at all* to 9 *very much*). Finally, we included two measures capturing the value of the goal to earn money. Like Aarts et al. (2004), we assessed people’s need for money by asking about people’s financial situation (“Do you have enough money in everyday life at the moment?”; 1 *not at all* to 9 *absolutely –* reversed). In addition, we measured greed for money with an altered dispositional greed scale (Seuntjens et al., 2016). Participants indicated on 9-point scales to what extent they agreed with the following items: “I think one can never have enough money,” “Actually, money is quite important to me” and “Even if I have a job, I always think about how I could earn more money” (Cronbach’s *α* = .74).

### Results

#### Manipulation check

In a first step, we checked with the spontaneous goal inference measure how often the goal of earning money was correctly reported in the earning money condition and incorrectly reported in the control condition. In the earning money condition, 83.8% reported the goal, compared to 8.8% in the control condition, *χ*^2^(1) = 90.50, *p* < .001, *Odds-Ratio* (*OR)* = 53.75. Next, we checked the effort manipulation. A 2 (goal: earning money vs. control) × 2 (effort: high vs. low) between-subjects ANOVA with perceived effort as dependent variable revealed the hypothesized main effect of effort (*F*(1,156) = 142.54, *p* < .001, *η_p_*^2^ = .477). Participants perceived the student’s behavior as more effortful when it was described as such – regardless of the goal condition (high effort: *M* = 6.12, *SD* = 1.34; low effort: *M* = 3.54, *SD* = 1.54). The interaction was not significant (*F*(1,156) = 1.93, *p* = .167, *η_p_*^2^ = .012), but there was an unexpected main effect of goal (*F*(1,156) = 18.39, *p* < .001, *η_p_*^2^ = .105): people who read about a student planning to volunteer in a community center perceived his/her behavior as more effortful (*M* = 5.23, *SD* = 2.03) than people who read about a student planning to work in a supermarket (*M* = 4.37, *SD* = 1.74).

#### Confirmatory analysis

To test if the student’s effort facilitates goal inference in the earning money condition, we submitted the data to a 2 (goal: earning money vs. control) × 2 (effort: high vs. low) between-subjects ANOVA with automatic goal inference as dependent variable. We found a significant two-way interaction of goal and effort (*F*(1,156) = 10.87, *p* = .001, *η_p_*^2^ = .065) and a significant main effect of goal manipulation (*F*(1,156) = 327.99, *p* < .001, *η_p_*^2^ = .678). There was no main effect of effort (*F*(1,156) = 0.67, *p *= .414, *η_p_*^2^ = .004). The effect of the goal manipulation was apparent in both effort conditions. Bonferroni-corrected comparisons revealed that participants inferred the goal of earning money more strongly when earning money was the actual goal (high effort: *M*_1_ = 16.25, *SD*_1_ = 3.50; low effort: *M*_2_ = 14.73, *SD*_2_ = 4.48) than when it was not (high effort: *M*_3_ = 3.08, *SD*_3_ = 2.29; low effort: *M*_4_ = 5.61, *SD*_4_ = 4.75; *F*(1, 156) = 226.32, *p* < .001, *d*_M1–M3_ = 4.445; *F*(1, 156) = 111.11, *p* < .001, *d*_M2–M4_ = 1.973). Furthermore and in line with our hypothesis, participants in the earning money condition inferred the goal marginally more often when the student showed high effort, compared to when he or she showed low effort (*F*(1, 156) = 3.07, *p* = .082, *d*_M1–M2_ = 0.380). The manipulation of effort in the control condition produced a significant effect in the opposite direction (*F*(1, 156) = 8.47, *p* = .004, *d*_M4–M3_ = 0.674; see Figure S1, panel A: https://osf.io/aw79e/).

#### Exploratory analyses

To see if need for money or greed influences goal inference, we calculated two regression models with goal (effect coded), effort (effect coded), need or greed (centered on the mean), and all interaction terms as predictors and automatic goal inference as criterion. In these analyses, none of the three-way interactions yielded an effect (see OSF). However, in the greed model, Goal × Greed predicted goal inference (*t*(152) = −2.64, *p* = .009, *b* = −1.76). The goal manipulation effect on goal inference was stronger for people low in greed (*M*-1*SD: t*(152) = 14.62, *p* < .001, *b* = 12.57) than for people high in greed (*M *+ 1*SD: t*(152) = 10.49, *p* < .001, *b* = 9.28). Furthermore, the greedier people were in the control condition, the more they inferred the goal of earning money (*t*(152) = 3.05, *p* = .003, *b* = 1.45). This relation was not significant in the earning money condition (*t*(152) = −0.67, *p* = .503, *b* = −0.32; see Figure S1, panel B: https://osf.io/aw79e/).

### Discussion

Our behavior descriptions successfully affected the perceived goal and perceived effort. More than 80% of the participants spontaneously reported the goal correctly in the earning money condition compared to less than 10% who falsely mentioned this goal in the control condition. Furthermore, participants perceived the work of the student who was willing to work extra time as more effortful. Crucially, effort facilitates goal inference when goal-relevant behavior is observed. This supports our main hypothesis that effort influences goal contagion because it moderates goal inference.

We also expected that the value of the goal does not alter goal inference. In line with this hypothesis, the effect of the goal manipulation on goal inference was not stronger the higher people’s need or greed was. It only seems to be the case that people with higher greed infer a goal to earn money to a greater extent even if the other person does not show any behavior pursuing such a goal – as it was the case in our control condition. It is also noteworthy that the need for money revealed a floor effect with two thirds of the participants scoring in the lower third of a 9-point scale. This indicates that need for money might not capture variation for goal value in our population. Even though null effects have to be interpreted cautiously, these results are in line with the hypothesis that the effect of goal value on goal contagion (Aarts et al., 2004) emerges because the value of the goal alters goal adoption – the second step in the goal contagion process.

## Study 1b: including implicit goal accessibility in the goal inference measure

Even though Study 1a confirms a successful manipulation of goal and effort, our measurement of goal inference seems to be suboptimal for several reasons. First, we could not use the planned coding of spontaneous goal inference and furthermore, the effect of effort in the goal-relevant condition was only a tendency. Second, one could argue that the inference measure contained no real implicit component, which is required for automatic processes. Third, one could question whether the product term of spontaneous and explicit goal inference is capturing the underlying psychological processes accurately. Fourth, to test the full two-step model of goal contagion it would be preferable to have a measure for the mediator goal inference that can be administered before the dependent variable goal adoption. However, administering the measure from Study 1a at this position would interrupt an automatic goal contagion effect, because we explicitly ask about the goal to earn money.

Therefore, we wanted to replicate the previous study with an additional *implicit goal accessibility* indicator. If participants automatically infer the goal of earning money, this goal and the concept of money should be rendered accessible (Aarts et al., 2004; Dijksterhuis & Aarts, 2010). Thus, the main goal of this study was to refine our automatic goal inference measure. We obtained data about spontaneous goal inference, explicit goal inference, and implicit goal accessibility, which – together – should be capturing automatic goal inference. Changing the measure for spontaneous goal inference from dichotomous to continuous enabled us to check whether all three load on a single *automatic goal inference* factor. Thus, while keeping the manipulation identical to Study 1a, we used a structural equation model to analyze the influence of goal and effort (and their interaction) on automatic goal inference, which was a latent variable indicated by implicit goal accessibility, spontaneous goal inference, and explicit goal inference.

### Method

We conducted Study 1b online by using Questback Unipark (QuestBack GmbH). The estimated time frame to complete this study was three weeks and we concluded it in two weeks. Participants from the student population first gave their informed consent and then answered basic demographic questions (age, gender, status as student).

#### Goal manipulation

Participants read the earning money vs. control texts that we already applied in Study 1a. The text appeared for 30 seconds on the screen. Subsequently, participants indicated whether they were able to read the text in the allotted time (“yes” or “no”).

#### Implicit goal accessibility

To assess Implicit goal accessibility for automatic goal inference, we presented participants with a word completion task directly after they read the goal manipulation texts (Dik & Aarts, 2007). Using a seemingly random letter, participants had to generate as many one-syllable words as possible within 30 seconds and type them into an answer field. All participants received the letter “G,” because our target word associated with the goal to earn money was “Geld” (“money” in German). The position of the target word within the string of produced words indicates the accessibility of the goal. Similar to Dik and Aarts (2007), we calculated participants’ relative score by dividing the position of the target word (counting backwards from the last word) by the total amount of produced words. For example, if “Geld” was mentioned on the second position in a list of seven words, participants would have received a score of 6/7 = 0.857. In that way, higher scores indicated higher goal accessibility. This measure was *z*-transformed.

#### Spontaneous goal inference

Participants then answered an open spontaneous goal inference question as before but with the addition “to list everything that comes to your mind.” Similar to Aarts et al. (2004, Study 6), we expected to receive a list of possible goals from the participants and applied the same recalculation and *z*-transformation as for the implicit goal accessibility score.

#### Explicit goal inference

Along with the distractor items, we adopted this explicit goal inference measure from Study 1a. As the other measures, it was *z*-transformed.

#### Love of money

Next, we measured goal value. Because the item assessing need for money used in Study 1a revealed a floor effect and greed for money alone might be too narrow of a definition for the varying value of earning money, we replaced both measures with the *importance of money* subscale from the love of money (LOM) questionnaire (Du & Tang, 2005). We added the word “earning” to each item to measure the value of earning money instead of the value of money itself. Our five items were “Earning money is important,” “Earning money is valuable,” “Earning money is good,” “Earning money is an important factor in the lives of all of us,” and “Earning money is attractive,” which were measured on 7-point scales (higher scores = more agreement).

#### Manipulation check and other measures

We assessed perceived effort with the same item as in Study 1a. In addition, we included two attention check questions regarding the text (“What will the person in the text be doing during the holidays?” and “When will the person start the described activity?” with specified responses from a list). We also asked participants whether they worked on the study undisturbedly and whether they themselves would include their data into our analysis (both “yes” or “no”). Thereafter, participants were thanked and offered a debriefing and a lottery participation via email.

### Sample, design, and power

As preregistered, we conducted a structural equation model analysis containing a 2 (goal: earning money vs. control) × 2 (effort: low vs. high) between-subject design with automatic goal inference as latent dependent variable. We analyzed the data with a structural equation modeling approach, reporting results from *Mplus 8* (Muthén & Muthén, 2010), but provide code for both *Mplus* and *lavaan* (Rosseel, 2017) in *R* (R Core Team, 2018) online (https://osf.io/cdp9v/).

We calculated the power for a liberal not-close-fit model with 9 degrees of freedom (21 variances and covariances; 12 parameters) and an alternative Root Mean Square Error of Approximation (RMSEA) of .08, compared to a Null RMSEA of .01 (R. MacCallum et al., 2010; Preacher & Coffman, 2006). At a power of 80%, this yielded a sample size of *N* = 300. However, we were also interested in the contrast between high and low effort in the earning money condition, which yielded an effect of *d* = .38 (*f* = .19) in Study 1a. To detect this contrast, a total sample size of 440 participants (220 per goal condition) was required to reach a power of 80% at *α* = .05 (Faul et al., 2007; Giner-Sorolla, 2018).

As indicated, we recruited participants via email and social media (Facebook groups). Eight hundred ninety-five persons clicked on the link to the study, 525 reached the final page, and 481 were interested in the lottery. As specified beforehand, participants were excluded from the confirmatory analysis if they reported that they had not read the manipulation text entirely within the allotted time, if they did not answer the two attention check items in the final questionnaire correctly, if they failed to complete the experiment (e.g. due to technical issues), or if they themselves encouraged us to do so. Based on these criteria, we had to exclude 101 participants from the analysis, leaving us with a final sample of *N* = 380, which laid between the two sample size calculations from above. A sensitivity analysis for the relevant low effort vs. high effort contrast in the goal group suggested that we could find a Cohen’s *d* of .41 with this sample size, which we deemed sufficiently close to the estimate above. As expected, participants were predominantly women and university students (*n*_female_ = 294, *n*_student_ = 364, *M*_age_ = 24.64 years, *SD*_age_ = 7.11).

### Results

All descriptive statistics can be found online on the OSF. As a manipulation check, we asked participants how much effort the main character in the stories would have to put in her or his work. A strong effect was found between the high effort (*M* = 7.23, *SD* = 1.41) and low effort condition (*M* = 3.60, *SD* = 1.76), *F*(1, 376) = 489.08, *p* < .001, *d* = 2.30.

#### Confirmatory analysis

As preregistered, we fitted several consecutive models, with goal and effort (both effect-coded) as well as their interaction predicting the latent variable goal inference, which consisted of implicit goal accessibility, spontaneous goal inference and explicit goal inference (all *z*-standardized) in different combinations (see Table 1). Our goal was to identify the model capturing a strong effect of our goal manipulation for goal inference, which was preferably moderated by effort.

**Table 1.** Tested models for Study 1b. Model 2 was selected based on sufficient fit criteria; see text for details.

	Model	*χ*^2^ (*df*), *p*	RMSEA [90%CI], *p*	SRMR, CFI, TLI, AIC, BIC (n-adjusted)
1		23.74 (9), .005	.07 [.03, 10], .182	.04,.96,.94, 4005.59, 4019.26
2		11.67 (5), .040	.06 [.01,.10], .309	.04,.98,.97, 2928.21, 2938.18
3		8.23 (3), .042	.07 [.01,.13], .235	.03,.99,.94, 4002.08, 4020.31
4		7.38 (2), .025	09 [.03,.15], .140	.02,.98,.95, 2880.48, 2888.07
5		No fit criteria available		
6		No fit criteria available		

G = goal manipulation, E = effort manipulation, AI = automatic inference, IMP = implicit goal accessibility, SPON = spontaneous goal inference, EXP = explicit goal inference.

Model 1 to 3 consisted of the two manipulations (goal vs. control and low effort vs. high effort) and their interaction (Goal × Effort) as IVs (see Table 1 for fit indices). The three indicators for the latent construct goal inference served as DV. Model 1 contained all three indicators and showed sufficient model fit, but the interaction was only marginally significant (*b* = 0.48, *z* = 1.67 *p* = .095) and implicit goal accessibility did not load on inference (*λ*_IMP_ = 0.03, *z *= 0.76, *p* = .445). Hence, implicit goal accessibility was eliminated for Model 2. In this model the Goal × Effort interaction remained marginally significant (*b* = 0.50, *z* = 1.76, *p* = .078), with both indicators loading sufficiently on inference (*λ*_SPON_ = 0.40, *z* = 12.42, *p* < .001; *λ*_EXP_ = 0.51, *z* = 9.84, *p* < .001; Cronbach’s *α* = .69). Moreover, most model fit criteria improved and according to a *χ*^2^-difference test Model 2 fitted significantly better (*χ*^2^_diff_ = 12.07, *df*_diff_ = 4). As can be seen in the simple effects in Table S1b (https://osf.io/6mye8/), we found large effects of the goal manipulation within the effort groups and considerably smaller effects between low and high effort within the goal groups (and this pattern was similar across all Models, see also https://osf.io/x8fz7/). Importantly, the slope between high and low effort reached marginal significance in the goal condition, *b* = 0.29, *z* = 1.85, *p* = .064, *d* = 0.29.

**Figure 1.**
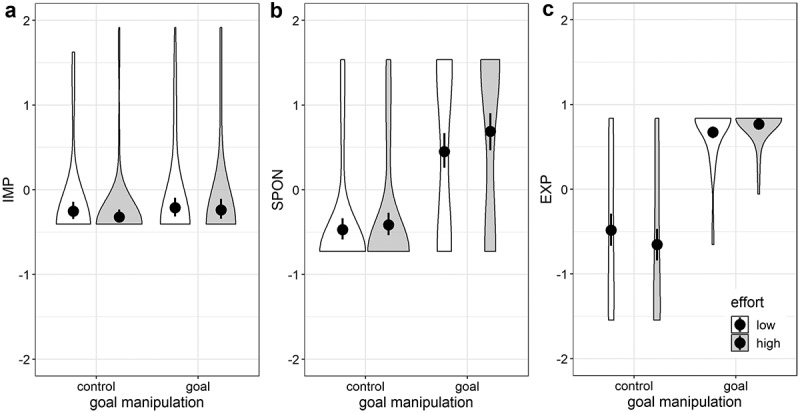
Violin plots for the three goal inference measures of Study 1b. Violins depict kernel density of the data. Black dots represent estimated means and whiskers represent the 95% confidence intervals. IMP (implicit goal accessibility), SPON (spontaneous goal inference) and EXP (explicit goal inference) are *z*-standardized.

Because in both Model 1 and 2 the interaction missed significance, we performed Model 3 with all three indicators as DVs (i.e. not as factor indicators of a latent variable). This model (patterns of the mean differences are shown in Figure 1) did not provide a better fit than Model 2 (*χ*^2^_diff_ = 3.44, *df*_diff_ = 2) and again none of the three Goal × Effort interactions were statistically significant.

To see if more parsimonious models would provide stronger effects on the key variables, we proceeded by eliminating effort and performed three more analyses similar to the previous three. Again, implicit goal accessibility did not load on inference in Model 4 (*λ*_IMP_ = 0.03, *z* = 0.80, *p* = .422), so we eliminated it. However, for the saturated Models 5 and 6 we did not identify considerably different effects of the goal manipulation on the latent variable (*b* = 2.49, *z* = 8.31, *p* < .001, *d* = 0.95) or on the three variables separately. We did find strong effects of goal on explicit goal inference (*b* = 1.29, z = 17.99, *p* < .001, *d* = 4.93) and spontaneous goal inference (*b* = 1.00, *z* = 10.43, *p* < .001, *d* = 1.27), but not on implicit goal accessibility (*b* = 0.15, *z* = 1.41, *p* = .160, *d* = 0.15), so we decided to stick to the two-indicator solution of Model 2.

#### Exploratory analyses

For the implicit measure, several participants came up with one-syllable words that were indirectly related to money. Those words were “gold” and “greed” (German: “Gold” and “Gier”). We also coded the position of those words and reran the analysis for Model 1. In this model, factor loadings remained unreliable for implicit goal accessibility (*λ* = −0.01, *z* = −0.22, *p* = .829) and we did not find larger effects for the interaction (*b* = 0.48, *z* = 1.70, *p* = .089).

We also collected data concerning participant’s LOM, which indicates personal goal value. We reran the analysis for Model 2, but with Goal × Effort and Goal × LOM as a partially latent interaction (Kline, 2011, Chapter 12). All indicators loaded significantly on the latent variable LOM (all *λ*s ≥ 0.52, *z*s ≥ 6.82, *p*s ≤.001, Cronbach’s *α* = .82), which in turn showed a statistically significant effect on inference (*b* = 0.171, *z* = 2.07, *p* = .038). Also, the LOM × Goal interaction (*b* = −0.294, *z* = −2.07, *p* = .076) indicated a slightly negative effect in the goal group with higher appreciation of money.

### Discussion

The primary aim of this study was to find a reliable measure for goal inference. We partially achieved this goal using a continuous version of the spontaneous goal inference measure and explicit goal inference measure as indicators. However, implicit goal accessibility did not bring about the expected loading as an implicit component of goal inference. Moreover, it was not predicted by the manipulations neither as part of the latent inference variable nor individually, as depicted in Figure 1 (panel A).

The apparent advantage of our implicit goal accessibility variable was that its operationalization as word completion task could be easily integrated in a complex experimental design as we have in Study 2. This way, it would have stood out from other, often more laborious setups employing cognitive measures like lexical decision tasks or dot probe paradigms. A downside of these other measures is that participants would be much longer involved, which may be detrimental to the goal manipulation, especially when behavioral measures yet have to follow. However, the word completion task did not detect differences across conditions.

The apparent floor effect (i.e. “money” was almost never mentioned) raises the question if this kind of word completion task leaves participants too many options for free associations. Possibly, words more common than “Geld” in the German language came earlier to their minds or earlier words influenced which words participants came up later with (e.g. coming up with a verb first might lead to another verb). Either way, the few times “Geld” did occur, it did not show a pattern across groups. Because false-negative findings can nevertheless occur, we kept it in our design for Study 2 as we preregistered, but did not focus on it in the confirmatory analysis.

A second result of Study 1b is that our effort manipulation yielded the expected simple effect only in marginally significant fashion despite utilizing a sample twice as large as in Study 1a. This is notable because if an effect truly exists, one would expect it to become more apparent in larger samples. Nonetheless, we decided to keep effort and directed our attention on the Goal × Effort interaction in the main study as the respective Model 2 showed the best model fit compared to other models. In Study 2, we tested the two moderators’ effort and personal goal value within the two-step model of goal contagion.

## Study 2: influence of effort and goal value on goal adoption

Using a structural equation model approach (Breitsohl, 2018), we investigated if the path from the goal manipulation (earning money vs. control) on automatic goal inference was moderated by effort and the path from automatic goal inference on goal adoption by goal value. Based on the results of Study 1a/b, we expected a main effect of the goal manipulation on automatic goal inference, but hesitated to expect a goal by effort interaction. However, effort was kept in the model as indicated in the preregistration.

**Figure 2.**
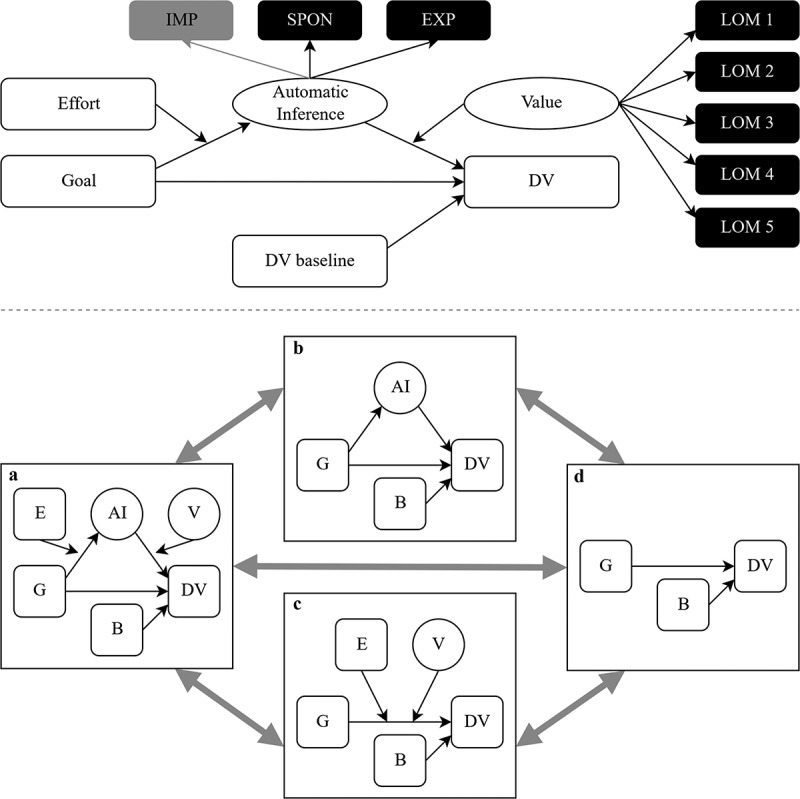
Preregistered conceptual model and model comparisons. Upper panel depicts the full model with the two-step process of goal contagion; note: IMP = implicit goal accessibility (not part of the tested model in Study 2), SPON = spontaneous goal inference, EXP = explicit goal inference, LOM = love of money; lower panel depicts model comparisons, note: the full model (a) will be compared to the less complex models (b) and (c) and the least complex model (d), variables in squares are observed variables, variables in circles are latent variables, G = goal manipulation, E = effort manipulation, AI = automatic inference, V = value, B = baseline, DV = dependent variable.

Within this model, we further tested if the effect of the goal manipulation on goal adoption was mediated by automatic goal inference. Baseline measures for the dependent variables were included as covariates. The full conceptual model is depicted in Figure 2 (upper panel).

### Method

We conducted Study 2 in two sessions. The first session was online, using Questback Unipark (QuestBack GmbH), and the second session was in the lab using Questback Unipark and PsychoPy, version 1.85 (Peirce, 2007). This study took approximately six months to be completed, which was longer than the anticipated 10 weeks. This is largely due to the conservative exclusion criteria (see below), which resulted in the necessity to oversample substantially. We recruited from the local universities’ student population. All materials were presented on a computer and are accessible via the OSF. In the first session, we measured goal value. The main part of the study took place in session two. The procedure in Session two followed Studies 1 a/b closely, but we added two additional tasks to measure goal adoption. We measured all three automatic goal inference indicators (implicit goal accessibility, spontaneous goal inference, and explicit goal inference). To measure implicit goal accessibility, we administered the word completion task between the manipulation and the tasks assessing goal adoption, whereas spontaneous goal inference and explicit goal inference were assessed after the goal adoption tasks. A final questionnaire at the end of the study included manipulation checks, demographics, and additional questions.

#### Session one

In the first part of the study, we measured goal value with the LOM questions from Study 1b along with some demographics (age, gender, student status) and distractor questionnaires (borrowed from Boer, 2013, Thomas et al., 2015), which we did not analyze. In the previous studies, we measured goal value after the manipulation, but there was a risk that the moderator would be influenced by the manipulation itself. Participants might read about a person trying hard to earn money, infer and adopt this goal and subsequently place more value on the goal while it is activated. We therefore decided to separate questions about goal value from the rest of the study. There were at least three days in between Session one and Session two, so that participants would not be influenced by the goal value measure.

#### Session two

During the second part of the study, we informed participants upon arrival that “the study contains several parts which will all take place at the computer. The exact number of tasks involved is flexible and depends on how far you will get in the allotted time frame. All of this will be explained in the instructions.” This cover story was necessary for our goal adoption measure (for a similar procedure see Aarts et al., 2004). Participants sat in front of one of several computer workstations, separated by partitions. They wore acoustic earmuffs over the course of the study.

Materials and measures for the goal and effort manipulation, implicit goal accessibility, and spontaneous as well as explicit goal inference were the same as in Study 1b.

#### Goal adoption

We measured goal adoption in two ways. In our first measure, we analyzed how quickly participants worked through a simple character search task to reach an opportunity for goal fulfillment. Therefore, participants were informed that the next task was about to start and if sufficient time was left by the end of this task, participants would get “the opportunity to earn some additional income” (in German: “Möglichkeit auf einen Zusatzverdienst”) in an extra task. Similar to the task used by Aarts et al. (2004), the character search task is an easy mouse-clicking task that lasts about 2 minutes. We instructed participants to click on all characters of one predefined kind in a 5 × 5 grid of different characters. The task encompassed 15 pages with four characters to find on each page. Participants proceeded automatically from page to page. The overall working time in seconds served as dependent variable (*speed*). We took a baseline measure of participant’s performance (*speed_base*) over 5 pages (plus one test trial beforehand) in the beginning of the experimental session, which we included as covariate in the design. As in the original study by Aarts et al. (2004, Study 1), we also asked participants whether it was their intention to work quickly on the character search task after this task was finished. They answered this question on a 9-point scale (from 1 “not at all” to 9 “absolutely”).^3^

**Figure 3.**
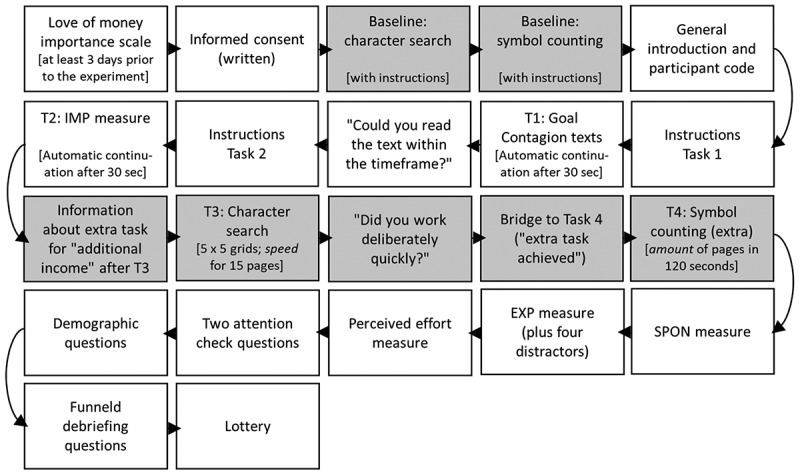
Experimental set-up for Study 2. Tasks in grey took part in PsychoPy, other tasks took part in Unipark (see materials https://osf.io/zgktj/).

Our second measure of goal adoption was participants’ performance in the extra task. All participants were informed that there was enough time left to work on the extra task for additional income. Specifically, we told them that the better they performed on the upcoming symbol counting task, the more tickets they would win and the higher their chances would be to receive an additional cash prize of 50€ (~60$), determined by a raffle.^3^ In this task, participants had to count the number of identical symbols in a cloud of jumbled symbols (squares, circles and triangles) on the left hand side of the screen as fast as possible and to write the numbers in text fields on the right hand side of the screen. Numbers ranged between 5 and 9. The next page appeared automatically once all numbers were filled in correctly. The number of pages completed within two minutes served as indicator of participants’ performance. Completing one page took about 10 seconds and we programed 20 pages in total (so participants’ score could range from 0 to 20). Better performance and therefore higher scores indicated greater adoption of the goal to earn money because performance determined the likelihood to win the cash price. Again, we took a baseline measure of participant’s performance (number of pages completed) over 45 seconds (including a one-page test trial) in the beginning of the experimental session, which we included as covariate in the design. The full experimental set-up is depicted in Figure 3.

#### Manipulation check and further measures

In addition to the questions of Study 1b, we inquired participants’ suspicion with three questions on three consecutive pages. We asked whether they thought the parts of the Study were connected (“yes” or “no”), whether they thought their behavior in the character search task and the symbol counting task was influenced by the text (“yes” or “no”) and how they thought they were influenced by the text (text field). Only if they directly indicate that they *worked quicker in part 2* or they wanted to *solve more pages in part 3*, we counted that as suspicion. Finally, participants were thanked and offered a debriefing via email.

### Power and participants

We estimated the required sample size for our structural equation model (which is equivalent to a partially latent structural regression model; Kline, 2011) in a conservative manner, where we assumed only one mediating variable (e.g. implicit goal accessibility) to obtain a conservative sample size estimate. Subtracting the parameters (24: 8 path coefficients, 4 indicator paths (one path will be set to 1) for goal value, 4 variances, and 8 error variances) from the known elements (66 variances and covariances without the latent variables) yields 42 degrees of freedom. We used those *df* along with an *α* error rate = .05, power of 80%, an alternative RMSEA = .05 and Null RMSEA = .01 (not-close-fit hypothesis, see R. C. MacCallum et al., 1996; Kline, 2011) and calculated the sample size via Preacher and Coffman (2006). This yielded a sample of 297 participants. To account for a potential dropout rate, we intended to add 10% more participants. For an equal distribution of participants across groups, we preregistered a sample size of *N* = 332 participants.

During data collection, a much higher dropout rate became apparent because some participants could not be matched across the two sessions based on their anonymous self-created code or because they did not answer the spontaneous goal inference question with regard to the text they had read. Even after we had 400 participants in the lab, we only had usable data from *N* = 228 participants. As we looked into the data at this point, we adjusted the *α* error via the R package *GroupSeq* (Lakens, 2014; Pahl, 2018) for multiple data peeking: the *p*-value for the moderated mediation effect should have been lower than .04 (with the effect in the expected direction) for the case that we would consider to stop data collection. However, this was not the case. Hence, we continued data collection to reach the full sample with the initial alpha-level.

In the end, 1461 persons clicked on the link to session one and 886 completed it. Then, 525 participants came to session two in the lab. After excluding participants according to the preregistered criteria (i.e. if they reported that they have not read the manipulation text entirely within the allotted time frame, if they did not answer the two attention check items in the final questionnaire correctly, if they could not complete the study due to technical issues, or if they indicated suspicion), we reached a final sample of *N* = 300. Our sample had a mean age of 22.04 years (*SD* = 4.26) and consisted mainly of women (*n* = 222) and students (*n* = 296)

### Results and discussion

Descriptive statistics and models can be found online (see https://osf.io/f9xc5/). As manipulation check, we checked if participants recognized the story character’s effort, which was the case between effort conditions (*F*(1, 296) = 266.32, *p* < .001, *d* = 1.88). Participants also indicated, whether they have worked intentionally quicker in the high effort condition than in the low effort condition in the speed task, which was largely not the case (*F*(1, 296) = 2.80, *p* = .096, *d* = 0.19).

#### Confirmatory analysis: the moderated-mediation model

We tested the main moderated-mediation model depicted in Figure 2. Because of the results of Study 1b, we included only spontaneous goal inference and explicit goal inference as latent variables to assess the mediator automatic goal inference for the confirmatory analyses. implicit goal accessibility was included in exploratory analyses. We expected that goal contagion is mediated by automatic goal inference and effort moderates the indirect path in the first step, whereas goal value moderates it in the second step. Thus, higher effort will facilitate automatic goal inference and thereby increase the goal effect on goal adoption. Goal value, on the other hand, will moderate the association between automatic goal inference and goal adoption. The higher participants’ individual perceived importance of earning money, the better automatic inference of the earning money goal will predict goal adoption. Like effort, LOM representing the value of the goal shall increase the effect of the goal manipulation on goal adoption. Goal adoption refers to two different and independently measured variables, namely participants’ speed in the character search task, and the number of completed pages by participants in the symbol counting task.

We conducted those moderated mediation analyses and preregistered model comparisons (Figure 2, lower panel; see supplementary spread sheets for statistics) using *MPlus* (Muthén & Muthén, 2010) as it is the most suitable program for latent variable interactions, which we have in our design (Maslowsky et al., 2015). The zero-order correlation between both outcome measures was large (*r* = .460, *p* < .001; Cohen, 1988) and remained significant when we controlled for the baseline measures (*r* = .189, *p* = .001), so we reduced the *α* error probability to .025 for the two analyses to account for familywise error.

**Figure 4.**
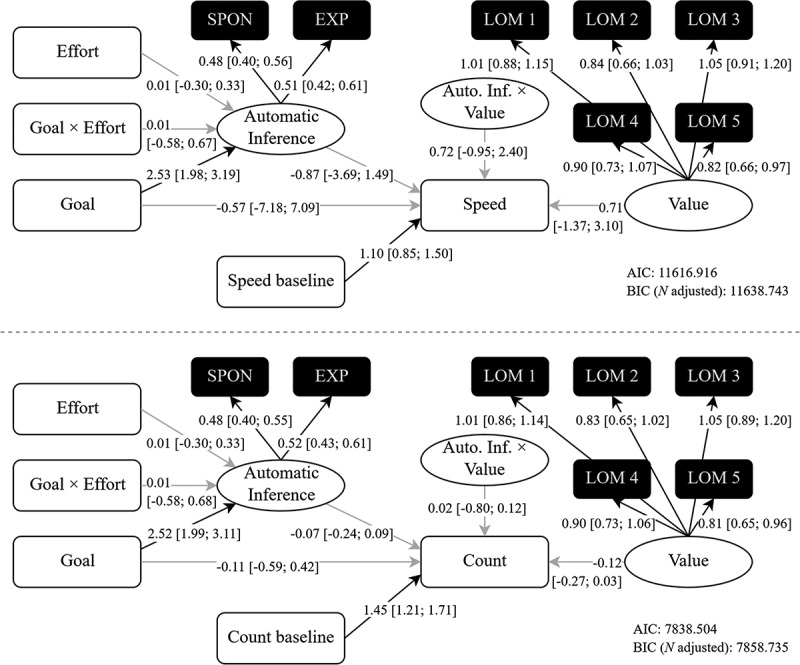
Confirmatory full statistical models. Coefficients with 95% CIs are unstandardized; DVs are *speed* (upper panel; higher scores = more speed) and symbol counting (*count*; lower panel; higher scores = more pages completed); black → indicates statistically significant effect (*p* <.025); grey → indicates non-significant effect; variances and error variances are omitted for simplicity.

Using participants’ speed and symbol counting as DVs in two full moderated mediation models, only two model fit parameters, the AIC and the BIC, could be produced. This was due to the continuous latent variables as both exogenous and endogenous variables and latent interactions in these models. Along with path coefficients and 95% bootstrap CIs, we present the results for both DVs in Figure 4. Interpretation will therefore focus on path coefficients. A summary of available model fit criteria and effects of interest per model can be found in Table 2.

**Table 2.** Tested models and effects of interest for Study 2.

Model	DV	AIC, BIC (n-adjusted)	Effect(s) of interest
A	Speed	11616.92, 11638.74	*b*_Goal×Effort → Inference_ = 0.01, *z* = 0.06, *p* =.956 *b*_Inference×Value → Speed_ = 0.72, *z* = 0.83, *p* =.407
	Pages	7838.50, 7858.74	*b*_Goal×Effort → Inference_ = 0.01, *z* = 0.04, *p* =.966 *b*_Inference×Value → Pages_ = 0.02, *z* = 0.40, *p* =.691
B	Speed *	3874.74, 3881.66	*b*_Goal → Inf. → Speed_ = −1.87, *z* = −0.62, *p* =.534
	Pages**	6503.04, 6508.89	*b*_Goal → Inf. → Speed_ = −0.19, *z* = −0.89, *p* =.372
C	Speed	8051.94, 8067.38	*b*_Goal×Effort → Speed_ = 2.00, *z* = 0.57, *p* =.568 *b*_Goal×Value → Speed_ = 3.29, *z* = 1.48, *p* =.140
	Pages	6473.75, 6489.18	*b*_Goal×Effort → Pages_ = 0.27, *z* = 1.03, *p* =.301 *b*_Goal×Value → Pages_ = 0.08, *z* = 0.50, *p* =.614
D	Speed	-	*b*_Goal → Speed_ = −0.08, *z* = −1.62, *p* =.105
	Pages	-	*b*_Goal → Pages_ = −0.30, *z* = −2.16, *p* =.031

Note: Additional fit indices for Model B.

* *χ*^2^(4) = 8.05, *p* =.090, *RMSEA* = 0.06, *CI*_90%_ [0.00; 0.12], *p*_RMSEA≤.05_ =. 338, *SRMR* =.03, *CFI* =.99, *TLI* =.98;

** *χ*^2^(4) = 6.52, *p* =.163, *RMSEA* = 0.05, *CI*_90%_ [0.00; 0.11], *p*_RMSEA≤.05_ =. 463, *SRMR* =.03, *CFI* =.99, *TLI* =.99.

First, for the DV speed, we did not find the anticipated effects in the Model A. Although we replicated the main effect of the goal manipulation on the goal inference (*b* = 2.53, *z* = 8.16, *p* < .001), there was no interaction with effort (*b* = 0.01, *z* = 0.06, *p* = .956). Inference and goal value did not show an interaction effect on speed (*b* = 0.72, *z* = 0.83, *p* = .407), rendering conditional indirect effects irrelevant. There was also no direct effect from the goal manipulation on participants’ speed of completing the task (*b* = −0.57, *z* = −0.16, *p* = .876). The baseline measure on speed (*b* = 1.10, *z* = 6.85, *p* < .001), as well as all indicators on their respective constructs (all *λ*s ≥ 0.48, *z*s ≥ 8.81, *p*s ≤ .001; Cronbach’s *α* for Goal Inference = .79, Cronbach’s *α* for LOM = .82) revealed significant effects even after a corrected alpha-error level.

**Figure 5.**
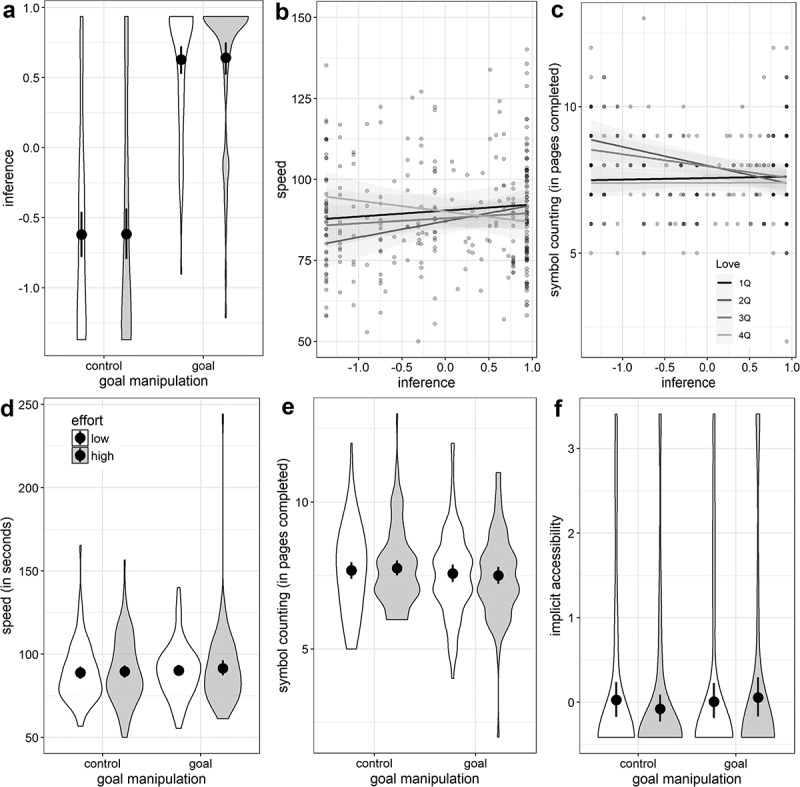
Additional analyses; panel A to C depict the raw effects of goal × effort on inference and inference × love of money in quartiles on speed and symbol counting, respectively; inference and love are based on a composite score of their respective items; panel D to F are exploratory analyses for speed, counting and implicit goal accessibility using a larger sample (*N* = 378); shades of the regression lines are 95% Cis; violins depict kernel density of the data; black dots represent estimated means and whiskers represent 95% CIs.

Those patterns were similar for Model A of the symbol counting task. Emphasizing the most important effects, there was no direct goal effect on the DV (*b* = −0.11, *z* = −0.43, *p* = .665), no interaction effect between goal and effort on inference (*b* = 0.01, *z* = 0.04, *p* = .966), and no interaction effect of inference and goal value on the number of pages solved in the task (*b* = 0.02, *z* = 0.40, *p* = .691). Patterns for the moderated paths are shown in Figure 5 (panel A to C) using composite scores.

None of the other models depicted in Figure 2 contained effects that we would describe as meaningful (all statistics: https://osf.io/cjtk4/). Firstly, the mediator model (Figure 2, lower panel, B) did not yield a mediation for neither speed, (*b* = −1.87, *z* = −0.62, *p* = .534; model comparison: ∆*AIC* = 7742.18, ∆*BIC*_n-adjusted_ = 7757.08), nor number of pages solved, (*b* = −0.19, *z* = −0.89, *p* = .372, model comparison: ∆*AIC* = 1335.46, ∆*BIC*_n-adjusted_ = 1349.85).

Secondly, as our mediator goal inference could be criticized for being incompatible with the theory because it does not contain an implicit element, we report results from the double-moderation model highlighted in Figure 2 (lower panel, C), which does not contain inference. In this model, the moderators effort and goal value should affect the goal contagion process for the behavioral outcome measure directly (see Aarts et al., 2004; Dik & Aarts, 2007). However, this was not case. For the DV speed (model comparison: ∆*AIC* = 3564.98, ∆*BIC*_n-adjusted_ = 3571.36), there was no interaction of either moderator (*b*_Goal×Effort_ = 2.00, *z* = 0.57, *p* = .568, *b*_Goal×Value_ = 3.29, *z* = 1.48, *p* = .140) and no main effect observed (*b*_Goal_ = −2.80, *z* = −1.54, *p* = .123, *b*_Effort_ = −2.46, *z* = −1.34, *p* = .180, *b*_Value_ = 0.88, *z* = 0.77, *p* = .440) beside a baseline effect (*b*_Base_ = 1.10, *z* = 7.10, *p* < .001). This was also true for the DV number of pages solved in the following count task, accounting for an adjusted alpha error (*b*_Goal×Effort_ = 0.27, *z* = 1.03, *p* = .301, *b*_Goal×Value_ = 0.08, *z* = 0.50, *p* = .614, *b*_Goal_ = −0.29, *z* = −2.17, *p* = .030, *b*_Effort_ = −0.12, *z* = −0.87, *p* = .383, *b*_Value_ = −0.12, *z* = −1.60, *p* = .110, *b*_Base_ = 1.48, *z* = 12.00, *p* < .001; model comparison: ∆*AIC* = 1364.76, ∆*BIC*_n-adjusted_ = 1369.56).

Thirdly, a similar pattern was observed for the simple multiple regression Model D, where the goal effect was not significant for speed (*b* = −0.08, *z* = −1.62, *p* = .105), and significant only without alpha correction for the DV symbol counting (*b* = −0.30, *z* = −2.16, *p* = .031). It has to be noted that effects for both DVs had a direction contrary to the hypothesis with control group participants having been faster on average and having completed more pages than participants in the goal group.

#### Exploratory analysis: the more powerful model

Beside exclusion criteria, the higher-than-expected dropout rate was caused by participants not remembering their codes from session one in which we assessed goal value and due to a misunderstanding of the spontaneous inference question in session two. Dropping these variables for exploratory purposes enables us to include *n* = 78 *(N* = 378) participants for an analysis of goal, effort and the baseline without other moderators or mediators. In this parsimonious factorial model, we did not find an effect on speed for either independent variable (*b*_Goal_ = −1.79, *z* = −1.12, *p* = .263, *d* = −0.12, *b*_Effort_ = −0.83, *z* = −0.52, *p* = .604, *d* = 0.05) or their interaction (*b* = 2.37, *z* = 0.74, *p* = .461). Similar small effects close to zero were observed for the number of pages solved (*b*_Goal_ = −0.16, *z* = −1.44, *p* = .151, *d* = −0.15, *b*_Effort_ = −0.06, *z* = −0.53, *p* = .596, *d* = −0.05, *b*_Goal×Value_ = 0.23, *z* = 0.97, *p* = .335). The baseline remained significant in both models (*b*_Speed_ = 1.16, *z* = 13.95, *p* < .001, *b*_Pages_ = 1.42, *z* = 14.43, *p* < .072). The mean differences are depicted in Figure 5 (panel D and E).

As we also collected data on the implicit measure that was utilized in Study 1b, we checked whether any evidence for an implicit inference could be found. However, we did not find an effect of the Goal × Effort interaction on implicit goal accessibility (*b*_Goal×Value_ = 0.23, *z* = 0.97, *p* = .335) and no main effect, either (*b*_Goal_ = −0.16, *z* = −1.44, *p* = .151, *d* = 0.15, *b*_Effort_ = −0.06, *z* = −0.53, *p* = .596, *d* = 0.05; see Figure 5, panel F). Furthermore, implicit inference was not associated with either outcome measure (*r*s ≤ .05, *p*s ≥ .441).

Finally, we wanted to evaluate whether our non-significant effects may be indicative for the null hypothesis being more likely than our alternative hypothesis. We used the goal manipulation and both DVs (controlled for the baseline) for a test of the null hypothesis via Bayes Factor. We employed the robust test statistics from the *t* test, which was 1.09 for speed and 1.40 for symbol counting. Those test statistics and the sample sizes of *N* = 378 (*n*_control_ = 193, *n*_goal_ = 185) were used in JASP’s summary statistics add on (JASP Team, 2019). We assumed a wide prior (Cauchy priori) to test the likelihood of the null hypothesis given the data. For both speed (*BF*_01_ = 6.87) and counting (*BF*_01_ = 4.71), we found moderate evidence that the null is more likely than the alternative hypothesis.

Taken together, the data of Study 2 did not yield evidence of the goal contagion process described in the introduction. Moreover, no evidence for an implicit inference process was found, either. As we applied conservative inclusion criteria, collected data from hundreds of participants and found moderate evidence for the null, we are confident in those results.

## General discussion

Everyday life shows us that people are getting inspired by other people: after learning about someone’s goals and aspirations, we sometimes adapt similar behaviors to pursue similar goals. A social-cognitive approach to this phenomenon is the effect of goal contagion (Aarts et al., 2004; Dik & Aarts, 2007). An implicit activation of a goal due to observing goal-directed behavior of another person would be a great base for inspiration, because goals guide behavior in a persisting and flexible way. However, the assumed two-step process of goal contagion is also vulnerable to the influences of moderators.

Thus, our primary interest was to better understand the effects of moderators that have been shown to be relevant in previous research (Aarts et al., 2004; Dik & Aarts, 2007). Those were the effort by the observed person to attain her goal and the observer’s individual value for the goal. We assumed that in the two-step model, the observed person’s effort strengthens the inference and adoption becomes more likely if the observer’s goal is in line with what s/he observes. With this Registered Report we set out to find evidence for the goal contagion effect within the full moderated-mediation model using the first goal that has been utilized in the context of the theory: earning money (Aarts et al., 2004, Study 1).

Across all three studies, there was strong evidence that participants could indeed infer the goal from the observed behavior. Participants always identified the goal of the person (spontaneous inference) and thought it was highly likely that the person wanted to earn money in the goal conditions (explicit inference), respectively. Hence, these two components could be treated as parts of the common factor goal inference. However, complications arose when we introduced an implicit measure to the same factor. The results of Study 1b were not indicative for an implicit accessibility: most participants did not list the word money and those who did were nearly equally distributed across conditions. This null-effect was replicated in Study 2 and persists in both studies even if the observed person showed high effort.

Effort should have moderated the path between the observation and inference. We found some evidence for this effect on the spontaneous and explicit inference components in Study 1a and b, but no evidence for such a moderation in Study 2. Importantly, even within the significant interaction, the crucial contrast between low and high effort in the goal condition was only marginally significant. Furthermore, there was no significant interaction on the implicit measure. Together, these mixed results do not substantiate our moderation hypothesis.

Thus, people correctly inferred the goal from the observed behavior. But did this translate into goal adoption as goal contagion would give reason to expect? This was not the case. In Study 2 we tested behavioral goal pursuit with two dependent variables in the moderated-mediation model: Participants had the opportunity to earn lottery tickets for a cash prize in task 2 (symbol counting task), if they worked fast enough in task 1 (speed). As higher speed in task 1 and more pages solved in task 2 indicate goal-directed behavior, they should have been associated with a successful inference. Moreover, goal value – participants’ LOM (Du & Tang, 2005), which was measured at least three days prior – should have moderated the path between goal inference and the DVs. Results of Study 2 for the two DVs did not turn out as hypothesized: neither inference, nor inference moderated by goal value showed an effect on the outcome measures. Furthermore, there was no main effect of the goal contagion manipulation on the two behavioral measures and, consequently, no support for adoption of the goal to earn money can be reported. These results raise several questions regarding our studies and the goal contagion effect.

First, one could ask whether our manipulation material was sufficient to elicit goal contagion. Given that previous research – both older (Aarts et al., 2004) and more recent (Laurin et al., 2016) – employed text-based material, we are confident that we followed established procedures. Importantly, our material was thoroughly pretested beforehand to ensure that the relevant goal was indeed recognized, which was approved before data collection started. Still it is possible that more vivid manipulation materials like videos could exert a more pronounced effect, although other studies of ours raise doubts to that claim (Brohmer et al., 2019a).

Second, one could ask whether the inference indicators were sufficient for our purposes. However, similar measures as spontaneous goal inference, explicit goal inference and implicit goal accessibility were employed by previous research on the same topic (Aarts et al., 2004; Dik & Aarts, 2007). Another critical issue is the position of the inference measure in the mediation model. Even though not uncommon (e.g. Jia et al., 2014), one might criticize that we measured spontaneous goal inference and explicit goal inference *after* the dependent variable in Study 2. We realized this order, because an explicit measure would bring the goal into conscious awareness and this might disturb an automatic goal contagion effect. We did not want to jeopardize the goal contagion effect – at the risk of reducing accuracy in capturing the mediation. Unfortunately, even without including the mediator in the model, we could not detect a goal contagion effect. Further, there are more profound issues with inference.

A closer look at these measures in the literature reveals some inconsistencies. For instance, evidence from a rather explicit inference measure of a goal (similar to our spontaneous goal inference measure) in Aarts et al. (2004, prestudy for Study 1, Study 6) was used to argue that the goal was activated in observers, implying that inference also worked on an implicit level and that goal contagion would occur. However, in another paper (Dik & Aarts, 2007, Experiment 2), it is argued against explicit inference (similar to our explicit goal inference measures) within the goal contagion process. Even though there was an effect on this explicit measure, this was not related to goal pursuing behavior. In our studies, we also show that both spontaneous and explicit measures are highly associated with our manipulation, but not with goal pursuit. In this regard, using explicit measures to show that participants recognize the goal correctly – even as a manipulation check, might be fruitless within goal contagion research, as they seem to be irrelevant for goal adoption.

Implicit measures, on the other hand, are manifold in the literature (e.g. Dik & Aarts, 2007; Hassin et al., 2005; Jia et al., 2014; Wessler & Hansen, 2016) and several authors argue in favor of them within the goal contagion process, which is sensible in theoretical terms. Therefore, it is surprising that few have tested a mediation model utilizing those measures (see exceptions Dik & Aarts, 2007; Jia et al., 2014). We used a variation of a word completion task twice and found two times no association with any variable in the model. Quite the contrary, data on our implicit measure clustered around zero across conditions. Unless we produced two false-negatives, our data strongly suggest that researchers interested in similar inference processes need to dedicate substantial effort to identify implicit measures that work well. This relates back to a wider issue of implicit measures we alluded to in the introduction.

Several implicit measures like the word completion task, the lexical decision task and others (see Eckerstorfer et al., 2019) are often employed in the literature (e.g. Dik & Aarts, 2007; Jia et al., 2014) to measure very different things: Foremost, they indicate construct accessibility (i.e. having a construct in mind due to internal processes or priming), but they are also used to measure goal inference (i.e. an observer processing someone’s goal), and goal activation/accessibility (i.e. having a goal cognitively activated to be pursued). Even though both goal inference and goal activation go hand in hand with construct accessibility, these are not the same. A goal is more than a construct and contains affective and motivational components. Thinking about money is not the same as thinking someone else wants money or wanting money oneself. However, none of the implicit measures seem to be able to differentiate between these three aspects. This is potentially why other authors have used the same implicit construct accessibility measure to assess goal adoption (e.g. Jia et al., 2014, Study 3) and goal inference (Dik & Aarts, 2007, Experiment 1). A differentiation, however, would not only be relevant in the context of goal contagion, but also for other goal activation processes (Kruglanski et al., 2002).

Our critical view of implicit measures is substantiated by other recent research in the field, which calls the validity of implicit measures into question (e.g. the Implicit Association Test, see Schimmack, 2019) or could not replicate effects that are based on assumptions of implicit activations (e.g. facial feedback, see Wagenmakers et al., 2016; professor priming, see O’Donnell et al., 2018). We would therefore suggest more direct replications of studies using other implicit measures, such as the lexical decision task (Jia et al., 2014; Wessler & Hansen, 2016). This step is essential for a substantial argument in favor of the theory, as there needs to be implicit measures found which can distinguish between mere construct accessibility, goal inference, and goal activation and which can be used to assess the mediator in the two-step goal contagion process.

Third, one could argue that our behavioral measures did not work and have to be improved. We relied on behavioral measures as they can show that a goal is not only inferred and accessible as a goal of another person, but also adopted by the observer. The downside of such a behavioral measure is that people do not always act on a goal – even though it is activated. We believe this downside is minimized within our experimental setup, as we provided participants an easy opportunity to pursue the goal to earn money. One might also have the concern that if a task for the dependent measure is too blatantly referring to an obvious goal, everyone might show the behavior that is relevant for the goal – regardless of the manipulation. Some ambiguity may be useful throughout the procedure (Loersch & Payne, 2011). In our studies, we avoided using the words “earning” and “money” with respect to the first dependent measure (speed), which was modeled after behavioral measures used in previous goal contagion studies (Aarts et al., 2004). However, for our second dependent variable (symbol counting), we clearly pointed out the opportunity to get lottery tickets for a cash prize, which might have been sufficient to activate the goal of earning money in everybody and thereby spoil the manipulation. However, we did not detect effects on any of the two dependent variables.

Fourth, one could ask if goal contagion actually exists. Goal contagion relies on a purely cognitive process. Maybe this viewpoint is too limited and one also has to consider motivational or emotional pathways that are important for goal activation (see Jia et al., 2014). For instance – and as we argue elsewhere (Brohmer et al., 2019a) – in order to get inspired for helpful behavior, one might first feel elevated by the observation in order to consider helping later. Likewise, a motivational boost by the observation of someone earning money might be required to truly stimulate observers pursuing the same goal. In this regard, the idea of implicit activations of associations that connect constructs and goals outside conscious awareness might not be sufficient for a goal contagion process. Just because earlier research on goal contagion has focused on a cognitive pathway and has rarely included emotional or motivational assessments does not mean that these alternative pathway do not exist or even sustain the goal contagion process.

It might also be that some aspects of the cognitive goal contagion process are not specified enough to enable replicability. For example, so far it is not quite clear whether goal contagion *could be* implicit and automatic or *has to be* implicit and automatic. On the one hand, the process seems to be highly plausible given its deduction from cognitive theories like implicit causal inference (Hassin et al., 2002), goal system theory (Kruglanski et al., 2002), implicit goal pursuit (Custers & Aarts, 2010), and associative networks (Shiffrin & Schneider, 1977, but see Amodio, 2018). On the other hand, our difficulties suggest some hidden procedures or unknown constraints. Maybe, we need to consider higher order moderations or have to identify subgroups of people and goals to be able to reliably show goal contagion. Moreover, by looking into the literature on goal contagion, we found a substantial publication bias (Brohmer et al., 2019b) and a lack of close replications of experimental paradigms. In other words, there are several published studies showing an effect, but these may be biased and none of those studies were replicated to substantiate, advance or question the theory (which defines an “undead theory” according to Chambers, 2017; Ferguson & Heene, 2012). We do think that inspiration is real, as everyday experiences show us. However, it is possible that the underlying processes are different and/or more complex than stressed by the seemingly straight-forward goal contagion process. In order to gain insight into mechanisms that impact goal contagion, we recommend that future research on this topic employs open science practices to ensure efficient theory building.

## Supplementary Material

Supplemental Material

## Data Availability

The data that support the findings of this study are openly available in the Open Science Framework at http://doi.10.17605/OSF.IO/EF59B, reference Brohmer et al. (2017).

## Notes

1. Please note a rounding error in the preregistration, where we reported *f* =.23.

2. In the preregistration, we planned to code spontaneous goal inference with 1, if participants report the goal to earn money, and with 0 otherwise. However, this resulted into an artificial skew in the distribution of the automatic goal inference variable, because the vast majority of participants in the control condition would score 0. We therefore decided to change the coding scheme to 1 and 2. All further analyses are based on that coding. Results for both coding schemes showed a similar pattern and can be found in the OSF.

3. Both the awareness item after the character search task and the instructions of the symbol counting task were in accordance with the preregistered materials, but deviated slightly from the descriptions in the preregistered manuscript, where we did not explicitly mention the awareness item (as it is not part of the confirmatory analysis) and used a different wording of the instructions.

## References

[cit0001] Aarts, H., Gollwitzer, P. M., & Hassin, R. R. (2004). Goal contagion: Perceiving is for pursuing. *Journal of Personality and Social Psychology*, 87(1), 23–37. 10.1037/0022-3514.87.1.23 15250790

[cit0002] Algoe, S. B., Haidt, J., & Gable, S. L. (2008). Beyond reciprocity: Gratitude and relationships in everyday life. *Emotion (Washington, D.C.)*, 8(3), 425–429. 10.1037/1528-3542.8.3.425 PMC2692821 18540759

[cit0003] Amodio, D. M. (2018). Social cognition 2.0: An interactive memory systems account. *Trends in Cognitive Sciences*, 23(1), 21–33. 10.1016/j.tics.2018.10.002 30466793

[cit0004] Bandura, A. (1977). *Social learning theory. Prentice-Hall series in social learning theory*. Prentice-Hall.

[cit0005] Bandura, A., Ross, D., & Ross, S. A. (1963). Imitation of film-mediated aggressive models. *The Journal of Abnormal and Social Psychology*, 66(1), 3–11. 10.1037/h0048687 13966304

[cit0006] Baron, R. M., & Kenny, D. A. (1986). The moderator–mediator variable distinction in social psychological research: Conceptual, strategic, and statistical considerations. *Journal of Personality and Social Psychology*, 51(6), 1173–1182. 10.1037/0022-3514.51.6.1173 3806354

[cit0007] Boer, D. (2013). Short Schwartz’s Value Survey in German (SSVS-G). Document retrieved from Goethe University Frankfurt. https://www.goethe-university-frankfurt.de/51799161/ssvsg_scale.pdf

[cit0008] Breitsohl, H. (2018). Beyond ANOVA: An introduction to structural equation models for experimental designs. *Organizational Research Methods*, 12(1), 649–677. 10.1177/1094428118754988

[cit0009] Brohmer, H., Eckerstorfer, L. V., van Aert, R. C. M., & Corcoran, K. (2019b). Is there inspiration through observation? A meta-analysis on goal contagion. https://osf.io/preprints/xydgf/

[cit0010] Brohmer, H., Fauler, A., Floto, C., Athenstaedt, U., Kedia, G., Eckerstorfer, L. V., & Corcoran, K. (2019a). Inspired to lend a hand? Attempts to elicit prosocial behavior through goal contagion. *Frontiers in Psychology*, 10(1), 1–16. 10.3389/fpsyg.2019.00545 PMC6449693 30984055

[cit0011] Brohmer, H., Hoefler, A., Corcoran, K., Eckerstorfer, L. V., Spörk, R., & Macher, S. (2017). Earning Money Goal: Conceptual Replication and Extension of Aarts et al. (2004). 10.17605/OSF.IO/EF59B

[cit0012] Chambers, C. (2017). *The seven deadly sins of psychology: A manifesto for reforming the culture of scientific practice*. Princeton University Press.

[cit0013] Chartrand, T. L., & Bargh, J. A. (1996). Automatic activation of impression formation and memorization goals: Nonconscious goal priming reproduces effects of explicit task instructions. *Journal of Personality and Social Psychology*, 71(3), 464–478. 10.1037/0022-3514.71.3.464

[cit0014] Chartrand, T. L., & Lakin, J. L. (2013). The antecedents and consequences of human behavioral mimicry. *Annual Review of Psychology*, 64(1), 285–308. 10.1146/annurev-psych-113011-143754 23020640

[cit0015] Cialdini, R. B. (2003). *Influence: Science and practice* (4th ed.). Allyn & Bacon.

[cit0016] Cohen, J. (1988). *Statistical power analysis for the behavioral sciences*. Erlbaum.

[cit0017] Custers, R., & Aarts, H. (2010). The unconscious will: How the pursuit of goals operates outside of conscious awareness. *Science (New York, N.Y.)*, 329(5987), 47–50. 10.1126/science.1188595 20595607

[cit0018] Dijksterhuis, A., & Aarts, H. (2010). Goals, attention, and (Un)consciousness. *Annual Review of Psychology*, 61(1), 467–490. 10.1146/annurev.psych.093008.100445 19566422

[cit0019] Dik, G., & Aarts, H. (2007). Behavioral cues to others’ motivation and goal pursuits: The perception of effort facilitates goal inference and contagion. *Journal of Experimental Social Psychology*, 43(5), 727–737. 10.1016/j.jesp.2006.09.002

[cit0020] Dik, G., & Aarts, H. (2008). I want to know what you want: How effort perception facilitates the motivation to infer another’s goal. *Social Cognition*, 26(6), 737–754. 10.1521/soco.2008.26.6.737

[cit0021] Du, L., & Tang, T. L.-P. (2005). Measurement invariance across gender and major: The love of money among university students in People’s Republic of China. *Journal of Business Ethics*, 59(3), 281–293. 10.1007/s10551-004-6395-4

[cit0022] Eckerstorfer, L. V., Brohmer, H., & Corcoran, K. (2019, 2). *Should we be Using Implicit Tasks to Measure Goal activation? Perhaps not* [Poster presentation]. The Society for Personality and Social Psychology’s Annual Convention 2019, Portland, OR.

[cit0023] Faul, F., Erdfelder, E., Lang, A.-G., & Buchner, A. (2007). G*Power 3: A flexible statistical power analysis program for the social, behavioral, and biomedical sciences. *Behavior Research Methods*, 39(2), 175–191. 10.3758/BF03193146 17695343

[cit0024] Ferguson, C. J., & Heene, M. (2012). A vast graveyard of undead theories: Publication bias and psychological science’s aversion to the null. *Perspectives on Psychological Science*, 7(6), 555–561. 10.1177/1745691612459059 26168112

[cit0025] Giner-Sorolla, R. Powering your interaction. https://approachingblog.wordpress.com/2018/01/24/powering-your-interaction-2/* (Original work published 2018).

[cit0026] Gollwitzer, P. M., & Moskowitz, G. B. (1996). Goal effects on action and cognition. In E. T. Higgins & A. W. Kruglanski (Eds.), *Social psychology: Handbook of basic principles* (pp. 361–399). Guilford Press.

[cit0027] Gollwitzer, P. M., & Oettingen, G. (2012). Goal Pursuit. In R. M. Ryan (Ed.), *The Oxford handbook of human motivation* (pp. 208–231). OUP.

[cit0028] Ham, J., & Vonk, R. (2003). Smart and easy: Co-occurring activation of spontaneous trait inferences and spontaneous situational inferences. *Journal of Experimental Social Psychology*, 39(5), 434–447. 10.1016/S0022-1031(03)00033-7

[cit0029] Hassin, R. R., Aarts, H., & Ferguson, M. J. (2005). Automatic goal inferences. *Journal of Experimental Social Psychology*, 41(2), 129–140. 10.1016/j.jesp.2004.06.008

[cit0030] Hassin, R. R., Bargh, J. A., & Uleman, J. S. (2002). Spontaneous causal inferences. *Journal of Experimental Social Psychology*, 38(5), 515–522. 10.1016/S0022-1031(02)00016-1

[cit0031] JASP Team. (2019). JASP (Version 0.8.4) [Computer software]. JASP Team. http://jasp-stats.org/download/

[cit0032] Jia, L., Koh, A. H. Q., & Tan, F. M. (2018). Asymmetric goal contagion: Social power attenuates goal contagion among strangers. *European Journal of Social Psychology*, 48(5), 673–686. Advance online publication. 10.1002/ejsp.2360

[cit0033] Jia, L., Lee, L. N., & Tong, E. M. W. (2015). Gratitude facilitates behavioral mimicry. *Emotion (Washington, D.C.)*, 15(2), 134–138. 10.1037/emo0000022 25286071

[cit0034] Jia, L., Tong, E. M. W., & Lee, L. N. (2014). Psychological “gel” to bind individuals’ goal pursuit: Gratitude facilitates goal contagion. *Emotion (Washington, D.C.)*, 14(4), 748–760. 10.1037/a0036407 24749636

[cit0035] Kline, R. B. (2011). *Principles and practice of structural equation modeling* (3rd ed.). The Guilford Press.

[cit0036] Kruglanski, A. W., Shah, J. Y., Fishbach, A., Friedman, R., Chun, W. Y., & Sleeth-Keppler, D. (2002). A theory of goal systems. In M. P. Zanna (Ed.), *Advances in experimental social psychology* (Vol. 34, pp. 331–378). Academic Press. 10.1016/S0065-2601(02)80008-9

[cit0037] Lakens, D. (2014). Performing high‐powered studies efficiently with sequential analyses. *European Journal of Social Psychology*, 44(7), 701–710. 10.1002/ejsp.2023

[cit0038] Laurin, K. (2016). Interpersonal influences on goals: Current and future directions for goal contagion research. *Social and Personality Psychology Compass*, 10(11), 668–678. 10.1111/spc3.12289

[cit0039] Laurin, K., Fitzsimons, G. M., Finkel, E. J., Carswell, K. L., Lambert, N. M., Lambert, N. M., Lambert, N. M., Eastwick, P. W., Fincham, F. D., & Brown, P. C. (2016). Power and the pursuit of a partner’s goals. *Journal of Personality and Social Psychology*, 110(6), 840–868. 10.1037/pspi0000048 27281354

[cit0040] Locke, E. A. (1969). What is job satisfaction? *Organizational Behavior and Human Performance*, 4(4), 309–336. 10.1016/0030-5073(69)90013-0

[cit0041] Loersch, C., Aarts, H., Keith Payne, B., & Jefferis, V. E. (2008). The influence of social groups on goal contagion. *Journal of Experimental Social Psychology*, 44(6), 1555–1558. 10.1016/j.jesp.2008.07.009

[cit0042] Loersch, C., & Payne, B. K. (2011). The situated inference model: An integrative account of the effects of primes on perception, behavior, and motivation. *Perspectives on Psychological Science*, 6(3), 234–252. 10.1177/1745691611406921 26168515

[cit0043] MacCallum, R., Lee, T., & Brown, M. W. (2010). The issue of isopower in power analysis for tests of structural equation models. *Structural Equation Modeling: A Multidisciplinary Journal*, 17(1), 23–41. 10.1080/10705510903438906

[cit0044] MacCallum, R. C., Browne, M. W., & Sugawara, H. M. (1996). Power analysis and determination of sample size for covariance structure modeling. *Psychological Methods*, 1(2), 130–149. 10.1037//1082-989X.1.2.130

[cit0045] Maslowsky, J., Jager, J., & Hemken, D. (2015). Estimating and interpreting latent variable interactions: A tutorial for applying the latent moderated structural equations method. *International Journal of Behavioral Development*, 39(1), 87–96. 10.1177/0165025414552301 26478643 PMC4606468

[cit0046] Meltzoff, A. N., & Moore, M. K. (1989). Imitation in newborn infants: Exploring the range of gestures imitated and the underlying mechanisms. *Developmental Psychology*, 25(6), 954–962. 10.1037/0012-1649.25.6.954 25147405 PMC4137867

[cit0047] Morgenroth, T., Ryan, M. K., & Peters, K. (2015). The motivational theory of role modeling: How role models influence role aspirants’ goals. *Review of General Psychology*, 19(4), 465–483. 10.1037/gpr0000059

[cit0048] Moskowitz, G. B. (2002). Preconscious effects of temporary goals on attention. *Journal of Experimental Social Psychology*, 38(4), 397–404. 10.1016/S0022-1031(02)00001-X

[cit0049] Muthén, L. K., & Muthén, B. O. (2010). *Mplus user’s guide* (6th ed.). Muthén & Muthén.

[cit0050] O’Donnell, M., Nelson, L. D., Ackermann, E., Aczel, B., Akhtar, A., Aldrovandi, S., Andringa, R., Aveyard, M., Babincak, P., & Balatekin, N. (2018). Registered replication report: Dijksterhuis and van Knippenberg (1998). *Perspectives on Psychological Science*, 13(2), 268–294. 10.1177/1745691618755704 29463182

[cit0051] Pahl, R. (2018). GroupSeq: A GUI-based program to compute probabilities regarding group sequential designs. The Comprehensive R Archive Network. https://cran.r-project.org/web/packages/GroupSeq/index.html

[cit0052] Peirce, J. W. (2007). PsychoPy–psychophysics software in python. *Journal of Neuroscience Methods*, 162(1–2), 8–13. 10.1016/j.jneumeth.2006.11.017 17254636 PMC2018741

[cit0053] Preacher, K. J., & Coffman, D. L. (2006). Computing power and minimum sample size for RMSEA. Retrieved from quantpsy. http://www.quantpsy.org/rmsea/rmsea.htm

[cit0054] QuestBack GmbH. Unipark. QuestBack GmbH. http://www.unipark.com/en/

[cit0055] R Core Team. (2018). *R: A language and environment for statistical computing*. R Foundation for Statistical Computing. https://www.R-project.org

[cit0056] Ross, L. (1977). The intuitive psychologist and his shortcomings: Distortions in the attribution process. In L. Berkowitz (Ed.), *Advances in experimental social psychology* (pp. 173–220). Academic Press.

[cit0057] Rosseel, Y. (2017). Package ‘lavaan’. The Comprehensive R Archive Network. https://cran.r-project.org/web/packages/lavaan/lavaan.pdf

[cit0058] Schimmack, U. (2019). The implicit association test: A method in search of a construct. *Perspectives on Psychological Science*. 10.1177/1745691619863798 31647752

[cit0059] Seuntjens, T. G., van de Ven, N., Zeelenberg, M., & van der Schors, A. (2016). Greed and adolescent financial behavior. *Journal Of Economic Psychology*, 57(1), 1–12. 10.1016/j.joep.2016.09.002

[cit0060] Shiffrin, R. M., & Schneider, W. (1977). Controlled and automatic human information processing: II. Perceptual learning, automatic attending and a general theory. *Psychological Review*, 84(2), 127–190. 10.1037/0033-295X.84.2.127

[cit0061] Tang, T. L. ‐. P., Chen, Y. J., & Sutarso, T. (2008). Management decision. *Bad Apples in Bad (Business) Barrels*, 46(2), 243–263. 10.1108/00251740810854140

[cit0062] Thomas, D. C., Liao, Y., Ayçan, Z., Cerdin, J.-L., Pekerti, A. A., Ravlin, E. C., Stahl, G. K., Lazarova, M. B., Fock, H., Arli, D., & van de Vijver, F. (2015). Cultural intelligence: A theory-based, short form measure. *Zusammenstellung sozialwissenschaftlicher Items und Skalen*. 46(9):1099–118. 10.6102/zis248

[cit0063] Wagenmakers, E.-J., Beek, T., Dijkhoff, L., Gronau, Q. F., Acosta, A., Adams, R. B., Jr, Allard, E. S., Benning, S. D., Blouin-Hudon, E.-M., & Bulnes, L. C. (2016). Registered replication report: Strack, Martin, & Stepper (1988). *Perspectives on Psychological Science*, 11(6), 917–928. 10.1177/1745691616674458 27784749

[cit0064] Weingarten, E., Chen, Q., McAdams, M., Yi, J., Hepler, J., & Albarracín, D. (2016). From primed concepts to action: A meta-analysis of the behavioral effects of incidentally presented words. *Psychological Bulletin*, 142(5), 472–497. 10.1037/bul0000030 26689090 PMC5783538

[cit0065] Wessler, J., & Hansen, J. (2016). The effect of psychological distance on automatic goal contagion. *Comprehensive Results in Social Psychology*, 1(1–3), 51–85. 10.1080/23743603.2017.1288877 29098177 PMC5644154

