# Supplementary material for: When your goals inspire my goals: the role of effort, personal value, and inference in goal contagion
Source: Compr Results Soc Psychol. 2020 Jun 8;4(1):78–108. doi: 10.1080/23743603.2020.1767502 (PMC7909217; doi:10.1080/23743603.2020.1767502)
Supplement: Supplemental Material [file RRSP_A_1767502_SM7881.docx]

**Supplementary Materials**

*Below, you can find all goal priming texts (male protagonist version) for Study 1a, 1b and 2 in German and in English. Note that the code in parenthesis corresponds with the code on the questionnaires.*

*All other materials are available here:* <https://doi.org/10.17605/OSF.IO/EF59B>

**Control, high effort (1a)**

Nach dem Ende des Semesters stehen für Stefan nun die Ferien vor der Tür. Für die nächsten vier Wochen hat er ein ehrenamtliches Praktikum in einem Freizeitzentrum bekommen. Bei dem Praktikum wird Stefan einiges zu tun haben: Er wird den ganzen Tag Schüler betreuen und Freizeitprogramme organisieren. Wahrscheinlich wird er viele Überstunden machen und wenn möglich auch an Wochenenden arbeiten. Das Praktikum startet nächsten Montag.

Stefan has finished his semester, and now holidays are just around the corner. In the upcoming four weeks, he is going to volunteer as an intern in a leisure center. During that time, there are many things to do: he will supervise pupils and organize their leisure time activities full-time. Probably he will work overtime and – if possible – also during weekends. The internship will begin next Monday.

**Money, high effort (2a)**

Nach dem Ende des Semesters stehen für Stefan nun die Ferien vor der Tür. Für die nächsten vier Wochen hat er einen saisonalen Studentenjob in einem Einkaufszentrum bekommen. Bei dem Job wird Stefan einiges zu tun haben: Er wird den ganzen Tag Regale einräumen und bei Lieferungen aushelfen. Wahrscheinlich wird er viele Überstunden machen und wenn möglich auch an Wochenenden arbeiten. Die Arbeit startet nächsten Montag.

Stefan has finished his semester, and now holidays are just around the corner. In the upcoming four weeks, he is going to work as a temporary employee in a mall. During that time, there are many things to do: he will stock up shelves and assist during deliveries full-time. Probably he will work overtime and – if possible – also during weekends. The job will begin next Monday.

**Control, low effort (1b)**

Nach dem Ende des Semesters stehen für Stefan nun die Ferien vor der Tür. Für die nächsten vier Wochen hat er ein ehrenamtliches Praktikum in einem Freizeitzentrum bekommen. Bei dem Praktikum wird Stefan nicht viel zu tun haben: Er wird halbtags Schüler betreuen und Freizeitprogramme organisieren. Er wird sicher keine Überstunden machen und auf keinen Fall am Wochenende arbeiten. Das Praktikum startet nächsten Montag.

Stefan has finished his semester, and now holidays are just around the corner. In the upcoming four weeks, he is going to volunteer as an intern in a leisure center. During that time, there are not too many things to do: he will supervise pupils and organize their leisure time activities part-time. Probably he will not work overtime and certainly not during weekends. The internship will begin next Monday.

**Money, low effort (2b)**

Nach dem Ende des Semesters stehen für Stefan nun die Ferien vor der Tür. Für die nächsten vier Wochen hat er einen saisonalen Studentenjob in einem Einkaufszentrum bekommen. Bei dem Job wird Stefan nicht viel zu tun haben: Er wird halbtags Regale einräumen und bei Lieferungen aushelfen. Er wird sicher keine Überstunden machen und auf keinen Fall am Wochenende arbeiten. Die Arbeit startet nächsten Montag.

Stefan has finished his semester, and now holidays are just around the corner. In the upcoming four weeks, he is going to work as a temporary employee in a mall. During that time, there are not too many things to do: he will stock up shelves and assist during deliveries part-time. Probably he will not work overtime and certainly not during weekends. The job will begin next Monday.
